# Molecular responses of genetically modified maize to abiotic stresses as determined through proteomic and metabolomic analyses

**DOI:** 10.1371/journal.pone.0173069

**Published:** 2017-02-28

**Authors:** Rafael Fonseca Benevenuto, Sarah Zanon Agapito-Tenfen, Vinicius Vilperte, Odd-Gunnar Wikmark, Peet Jansen van Rensburg, Rubens Onofre Nodari

**Affiliations:** 1 Department of Crop Science, Federal University of Santa Catarina, Florianópolis, Brazil; 2 Genøk Center for Biosafety, Tromsø, Norway; 3 Department of Engineering & Natural Sciences, Western Norway University of Applied Siences, Sogndal, Norway; 4 Unit for Environmental Science and Management, North-West University, Potchefstroom, South Africa; 5 Human Metabolomics, North-West University, Potchefstroom, South Africa; Henan Agricultural University, CHINA

## Abstract

Some genetically modified (GM) plants have transgenes that confer tolerance to abiotic stressors. Meanwhile, other transgenes may interact with abiotic stressors, causing pleiotropic effects that will affect the plant physiology. Thus, physiological alteration might have an impact on the product safety. However, routine risk assessment (RA) analyses do not evaluate the response of GM plants exposed to different environmental conditions. Therefore, we here present a proteome profile of herbicide-tolerant maize, including the levels of phytohormones and related compounds, compared to its near-isogenic non-GM variety under drought and herbicide stresses. Twenty differentially abundant proteins were detected between GM and non-GM hybrids under different water deficiency conditions and herbicide sprays. Pathway enrichment analysis showed that most of these proteins are assigned to energetic/carbohydrate metabolic processes. Among phytohormones and related compounds, different levels of ABA, CA, JA, MeJA and SA were detected in the maize varieties and stress conditions analysed. In pathway and proteome analyses, environment was found to be the major source of variation followed by the genetic transformation factor. Nonetheless, differences were detected in the levels of JA, MeJA and CA and in the abundance of 11 proteins when comparing the GM plant and its non-GM near-isogenic variety under the same environmental conditions. Thus, these findings do support molecular studies in GM plants Risk Assessment analyses.

## 1. Introduction

Recurrent advances in molecular techniques and scientific knowledge have resulted in the ability to genetically manipulate many organisms, especially the development of genetically modified (GM) plants. And because GM plants are regulated in most countries, risk assessment (RA) and equivalence criteria to assess the safety of GM crops have been conducted during the past few years [1–7]. The concept of ‘substantial equivalence’ can be defined as the close nutritional and elemental similarity between a GM crop and a non-GM near-isogenic variety. It has often been used to claim that GM crops are as safe and nutritious as currently consumed plant-derived foods [8]. However, the parameters, criteria and analyses used to declare a GMO substantially equivalent are unclear and reduced to a limited set of variables, such as the total amounts of carbohydrates, proteins and minerals. In addition, pesticide residues can become part of the composition of a herbicide-tolerant GM plant, and they may add toxic properties to the final plant product, either alone or by affecting plant metabolism [9]. Nonetheless, the identification of such pesticide residues is overlooked in compositional analyses [10]. Therefore, one of the most reliable ways to detect unintended consequences of genetic modification is through a more detailed survey of molecular components [11].

Herbicide use has been steadily increasing, particularly after the release of herbicide-tolerant GM crops [12] which encompass approximately 80% of GM crops, and are able to accumulate residues without dying in order to facilitate weed management [13]. By definition, herbicides are chemicals that impair plant development and, therefore, are used to eliminate many different weeds [14]. Herbicide application is considered one of the major abiotic stressors of both weed and herbicide-tolerant GM plants, leading to stress response pathways in exposed plants, thus affecting gene expression and physiology [15–17]. Stress is defined as any external abiotic or biotic constraint that reduces a plant’s ability to convert energy into biomass [18]. In most cases, low crop productivity can be attributed to various abiotic stresses, including high salinity, drought, cold, and heat, all of which negatively influence the survival, biomass production, and yield of major crops [19–21]. Specifically, drought stress can be defined as any reduction in the amount of water available for the crop, regardless of irrigation, which reduces its productivity below expected yields under adequate water supply. Drought is a common recurring phenomenon and its effects are extremely complex [22, 23]. Water shortage leads to reduced growth for the entire plant, especially in the shoots, as well as alterations on the regulation of gene expression products, such as hormones, transcripts and proteins [24–27].

Changes at transcript level are not necessarily reflected by corresponding changes in plant proteome [28,29]. Thus, thorough investigations regarding changes in plant proteome submitted to stress conditions are crucial, since they directly indicate plant stress responses. Unlike the genome, which is expected to be constant for an organism, the proteome is highly dependent on environmental influences, such as abiotic stresses [4]. Profiling tools (e.g. proteomics) applied to the molecular characterization step can assist on the identification of potentially hazardous products, such as toxins, allergens and antinutrients. In addition, with the emergence of new GMO types, broader use of molecular profiling tools are required for reliable pre-market RA [30]. Moreover, plant hormones play central roles in the ability of plants to adapt to changing environments and the resultant abiotic stress. Phytohormones can also rapidly alter gene expression by inducing or preventing the degradation of transcriptional regulators via the ubiquitin-proteasome system [31]. Furthermore, some of these plant hormones are the main mediators of plant defense mechanisms against stress, such as plant growth regulators (e.g. abscisic acid (ABA), ethylene, cytokinins (CK), auxin (IAA), gibberellin (GA), jasmonate (JA), salicylic acid (SA), among others) [31].

Generally, RA analyses do not include information on the response of GM plants exposed to different environmental conditions. Water deficit has been common in major growing regions where, at the same time, the application of herbicides is a common occurrence. Thus, it seems reasonable to assess the response of GM plants to adverse conditions, especially in light of climate change times. Therefore, to test our hypothesis that abiotic stress conditions lead to off-target effects in protein and metabolite levels in GM plants, we have performed a proteomic and metabolomic characterization of the herbicide-tolerant NK603 GM maize and its non-GM near-isogenic line, under distinct stress conditions, including drought and herbicide application.

## 2. Material and methods

### 2.1. Plant material and experimental design

In order to elucidate the responses of a genetically modified maize plant to water stress and herbicide application, two independent experiments were performed. Both experiments were carried out under identical growing conditions, except for the application of Roundup herbicide in the second experiment.

Experiment 1 aimed at comparing the herbicide-tolerant GM maize DKB 245 RR2 (transformation event NK 603; unique identifier MON-ØØ6Ø3-6 from Monsanto Company, glyphosate-based herbicide tolerance, Dekalb Brand Corn) and its near-isogenic non-GM counterpart DKB 245 under water stress. Seeds of these hybrids were previously germinated in a growth chamber (Eletrolab^™^ Model 202/3) set to 16 h light period and 25°C (± 2°C). Seedlings were placed in 8 L plastic pots filled with Plantmax HT substrate (Buschle & Lepper S.A), transferred to a greenhouse and watered daily, keeping substrate moisture at 125% (1.25 g H_2_O / g of soil) controlled by a soil tensiometer (WaterMeter MOD WS-76). The plants were kept in these conditions until they reached five leaves fully emerged (stage V5), then a subset of both varieties (GM and non-GM) were stressed by withholding irrigation, according to Vicent et al. [16] with modifications. The drought stress period was maintained until substrate moisture reached 25% (0.25 g H_2_0 / g of soil), approximately 15 days after water restriction. At this time, leaf samples were collected. Studies on plant response to water deprivation report a significant decrease in leaf conductance from the seventh or eighth day of water stress, indicating that the stress is being perceived by the plant [32, 33]. For this reason, plants in Experiment 1 were subjected to 15 days of water stress, allowing the opportunity for direct response to the stress condition.

Experiment 2 aimed at testing the influence of herbicide application on the herbicide-tolerant GM maize hybrid (NK603). This experiment was carried out similarly to Experiment 1, except that two sequential applications of the commercial herbicide Roundup Original (Monsanto do Brasil LTDA) were done in a subset of twelve plants. Herbicide application was performed following the manufacturer’s recommendation. The first application took place 15 days after plant emergence, and the second application was performed 30 days after emergence, both at doses of 2.0 L / ha in a concentration of 960 g of glyphosate (isopropylamine salt) per ha. Similar to Experiment 1, the drought stress period was maintained until substrate moisture reached 25%.

For both experiments, 12 plants per treatment were randomly sampled and separated into three groups of four plants each. The four plants of each group were pooled and were considered as one biological replicate (sample) of each treatment for the proteomic and plant hormone analysis. Then, leaf pieces were cut from each biological replicate, weighed, immediately placed in 3.8 ml cryogenic tubes, and frozen in liquid nitrogen. The samples were kept at -80°C until protein and hormone extraction.

### 2.2. Proteomic analysis

#### 2.2.1. Protein extraction and fluorescence hybridization

For two-dimensional (2D) Difference gel electrophoresis (DIGE) proteomic analysis, we followed the methodology of Agapito-Tenfen et al. [1]. Briefly, approximately 100 mg of each sample were ground in a mortar with liquid nitrogen and subsequently extracted and precipitated using phenol and methanol/ammonium acetate. Pellets were re suspended in a urea/thiourea buffer compatible with further fluorescent labelling (4% CHAPS (w/v), 5 mM PMSF, 7 M urea, 2 M thiourea and 30 mM Tris-base). Protein quantification was performed with the 2-D Quant Kit (GE Healthcare Bio-Sciences AB, Uppsala, Sweden). Eighty μg of each protein sample pool were labelled with 400 ρmol/μl of CyDye DIGE fluors (Cy3 and Cy5; GE Healthcare) and subsequently stored at -80°C. An internal standard labelled with Cy2 was used in every run for normalization. The internal standard was a mixture of equal amounts of each plant variety sample. After protein-fluor hybridization, lysine (10 mM) was used to stop the reaction and then mixed together for 2D-DIGE. Sample pairs were randomly selected for bi-dimensional electrophoresis runs.

#### 2.2.2. 2-D DIGE conditions

After protein labelling, samples were prepared for the isoelectric focusing (IEF) step. Strip gels of 24 cm and a linear pH range of 4–7 (GE Healthcare) were used. Strips were initially rehydrated with labelled protein samples (7 M urea, 2 M thiourea, 2% CHAPS (w/v), 0.5% IPG buffer (v/v) (GE Healthcare), 2% DTT). Strips were then processed using an Ettan IPGPhor IEF system (GE Healthcare) in a total of 35,000 Volts.h^-1^ and, subsequently, reduced and alkylated for 30 min under slow agitation in Tris-HCl solution (75 mM), pH 8.8, containing 2% SDS (w/v), 29.3% glycerol (v/v), 6 M urea, 1% DTT (w/v) and 2.5% iodocetamide (w/v). The 2D- DIGE conditions were performed as described by Weiss and Görg [32]. Gels were immediately scanned with a FLA-9000 modular image scanner (Fujifilm Lifescience, Dusseldorf, Germany). To ensure maximum pixel intensity between 60,000 and 90,000 pixels for the three dyes, all gels were scanned at a 100 μm resolution and the photomultiplier tube (PMT) voltage was set between 500 and 700 V. As described by Agapito-Tenfen et al. [2], we performed preparative gels for each plant variety in order to extract relevant spots. These were performed with a 450 μg load of total protein pools in 24 cm gels from each variety, separately, and stained with colloidal Coomassie Brilliant Blue G-250 (MS/MS compatible).

#### 2.2.3. Image analysis

The scanned gel images were transferred to the ImageQuant V8.1 software package (GE Healthcare) and appropriately cropped. Afterwards, the images were exported to ImageMasterTM 2D Platinum 7.0, version 7.06 (GE Healthcare), for comparative analysis. Each 2D-DIGE gel is loaded with treatment and control samples plus an internal standard control (equal mix of all samples of the experiment) and leabeled with a different fluorescence fluor. Therefore, the DIGE software uses a co-detection algorithm, which is capable of realizing simultaneous detection of protein spots labelled in different images, but present on the same gel and normalize it with the internal control. Thus, this co-detection significantly increases the accuracy of a comparative proteomic study, especially compared to traditional 2D-PAGE using Comassie Brilliant Blue. Thus, the simultaneous electropheresis of treatment and control samples, followed by normalization with the corresponding internal standard greatly minimizes technical variation and thus does not require technical replicate gels. In fact, technical replicates are represented by the internal standard control which is present in every DIGE gel.

#### 2.2.4. In-gel digestion and protein identification by MS/MS

Spots from preparative gels were excised and sent to the Proteomic Platform Laboratory at the University of Tromsø (Norway) for processing and analysis. They were subjected to in-gel reduction, alkylation, and tryptic digestion using 2–10 ng/μl trypsin (V511A; Promega) [34]. Peptide mixtures containing 0.5% formic acid were loaded onto a nano ACQUITY Ultra Performance LC System (Waters, Milford, MA, USA), containing a 5-μm Symmetry C18 Trap column (180 μm × 20 mm; Waters) in front of a 1.7-μm BEH130 C18 analytical column (100 μm × 100 mm; Waters). Peptides were separated with a gradient of 5–95% acetonitrile, 0.1% formic acid, with a flow rate of 0.4 μl/min eluted to a Quadrupole Time-of-Flight (Q-TOF) Ultima mass spectrometer (Micromass; Waters). The samples were run in data- dependent tandem MS mode. Peak lists were generated from MS/MS by Protein Lynx Global server software (version 2.2; Waters). The resulting ‘pkl’ files were searched against the NCBI protein sequence databases using Mascot Distiller, ver. 2.5.1.0 (Matrix Sciences; http://matrixscience.com). Data were searched against the NCBInr database, ver. 20151109, with Viridiplantae taxonomy (Green Plants; 3334509 sequences) and against ‘all entries’ and ‘contaminants’ (76068736 sequences; 27658295194 residues) for contamination verification. The following parameters were adopted for database searches: complete carbamidomethylation of cysteines and partial oxidation of methionines; peptide mass tolerance ± 100 ppm; fragment mass tolerance ± 0.1 Da; missed cleavages 1; and significance threshold level (P < 0.05) for Mascot scores (-10 Log (P)). Even though high Mascot scores could be obtained with significant values, a combination of automated database searches and manual interpretation of peptide fragmentation spectra was used to validate protein assignments. Molecular functions and cellular components of proteins were searched against the Encyclopedia of Genes and Genomes (KEGG) Orthology system database, release 69.0 2014 (http://kegg.jp/kegg/ko.html).

#### 2.2.5. Pathway enrichment analysis

Differentially abundant proteins were submitted to enrichment analysis by the online tool agriGO, v1.2 [35], using the Single Enrichment Analysis (SEA) category, with the following parameters: 1) Selected species: *Zea mays* ssp; 2) Statistical test method: Hypergeometric; 3) Multi-test adjustment method: Hochberg (FDR); 4) Significance level of 0.05; 5) Minimum number of 5 mapping entries; and 6) Gene ontology type: Plant GO Slim. Next, the online tool REVIGO [34] was used to remove the redundant Gene Ontology (GO) terms. Only significant GO terms (False Discovery Rate (FDR) values < 0.05) were used with the following parameters: 1) Allowed similarity: medium (0.7); 2) Database with GO term sizes: *Zea mays*; and 3) Semantic similarity measure: SimRel.

### 2.3. Plant hormone and related compounds

#### 2.3.1. Compound extraction

Samples were polled up to 40 mg of freeze-dried leaf tissue (biological replicates) for the extraction of phytohormones and related compounds. The leaf tissue was ground in liquid nitrogen until total spraying, according to Pan et al. [36]. Subsequently, 500 μl of extraction solution consisting of isopropanol, H_2_O and HCl (2:1:0.002, v/v/v) were added in each tube. Tubes were then placed on a shaker at 100 rpm for 30 min at 4°C. After stirring, 1 ml of dichloromethane solvent and 10 μl of 2-acetamidophenol (10 ppm) (internal standard) were added to each tube and stirred at 100 rpm for 30 min at 4°C. Samples were centrifuged at 13,000 g for 5 min at 4°C. After centrifugation, 900 μl of solvent present in the lower phase were transferred into a new tube using a Pasteur pipette. The solvent mixture was concentrated (not completely dried) using a nitrogen evaporator with nitrogen flow and then dissolved in 100 μl of methanol. Samples were kept at -20°C until analysis.

#### 2.3.2. Liquid chromatography—Tandem mass spectrometry

The quantitative analysis of phytohormones and related compounds was conducted at the Metabolomic Centre of the Department of Biochemistry (North-West University, South Africa). Quantification of phytohormones and related compounds, which belong to the main classes involved in plant defense-related metabolic pathways during stress, was performed for Salicylic Acid (SA), Jasmonic acid (JA), Methyl Jasmonate (MeJA), Abscisic acid (ABA), Indoleacetic acid (IAA) and Cinnamic acid (CA). Quantification was determined using reverse-phase liquid chromatography-tandem mass spectrometry with multiple reaction monitoring [36]. An Agilent 1290 Liquid chromatograph coupled to an Agilent 6460 triple quadrupole mass spectrometer was used. Chromatography was performed with an Agilent Zorbax Eclipse plus C18 (2.1 x 100 mm, 1.8 μm). The chromatographic separation started with 5% solvent B (methanol) and 95% solvent A (water and 0.1% formic acid). The percentage of solvent B was increased to 90% over 7 min. From 7 to 9 min, solvent B was maintained at 90% and then decreased after 9 min to reach 5% solvent B at 12 min. A post-run of 4 min was used to ensure column re-equilibration. A constant flow rate of 0.2 ml/min and a column temperature of 50°C were used.

The ESI source with Agilent Jet Stream technology was set in either positive or negative ionization for each of the two methods used. A gas temperature of 325°C, gas flow of 10 L/min and nebulizer pressure of 45 psi were used in both methods. Multiple reactions monitoring (MRM) transition of each analyte was optimized (Additional file 1). Three runs (technical replicates) were conducted for each biological pool, to inform the technical variation.

### 2.4. Statistical analysis

Multiple Co-Inertia Analysis (MCIA) was performed in order to explore the experimental quality and the main sources of variation in proteomic and plant hormone datasets simultaneously. For the proteomics experiment, one-way ANOVA was used to investigate differences at individual protein levels. Tukey test at P < 0.05 was used to compare the multiple means in the dataset. The calculations were performed on normalized spot volume ratios based on the total intensity of valid spots in a single gel. Differences at P < 0.05 were considered statistically significant. Statistical analyses were performed using ImageMasterTM 2D Platinum ver. 7.06 (GE Healthcare). For plant hormone quantitative analysis, one-way ANOVA and Tukey test were also used at statistical significance of P < 0.05. Because of non-normal distribution, the data were natural logarithm (LN) transformed. Fold changes are also presented in logarithm base 2 (Log2FC). Log2FC is the most widely used alternative transformation of the ratio since it has the advantage of producing a continuous spectrum of values and representing up- and downregulated compound values in a similar and reader friendly fashion [37].

## 3. Results and discussion

Proteomic profile and key metabolic compounds were analysed in comparison to the non-GM near-isogenic variety under the same experimental conditions aiming at exploring molecular responses of GM maize variety to drought and herbicide stress conditions. To facilitate the presentation of results, we have named each plant variety and its corresponding growing conditions as follows: Experiment 1—non-GM D (conventional hybrid under drought stress); non-GM (Conventional hybrid under normal growth conditions); GM D (GM hybrid under drought stress); GM (GM hybrid under normal growth conditions). Experiment 2—GM DH (GM under drought stress and herbicide application); GM D (GM hybrid under drought stress) (Table 1).

**Table 1:** Commercial maize varieties and treatments used in this study.

Variety commercial name	GM event name	Transgenes	Stress condition	Study designation
DKB 245	non-GM	-	Drought	non-GM D
DKB 245	non-GM	-	Control	non-GM
DKB 245 RR2	NK603	CP4EPSPS	Drought	GM D
DKB 245 RR2	NK603	CP4EPSPS	Control	GM
DKB 245 RR2	NK603	CP4EPSPS	Drought + Herbicide	GM DH

### 3.1. Experimental quality assessment using MCIA

MCIA has been recognized as an excellent tool for integrating the results of different "omics" techniques. It is an exploratory data analysis method that is able to provide a simple graphical representation that identifies the concordance between these multiple datasets [38]. It is noteworthy that one component of MCIA can be used to highlight the absence or presence of co-structure between the datasets, thereby permitting the choice of the strongest elements for the following analysis [38], and based on these merits, MCIA was applied to this exploratory analysis of proteomic and metabolic studies in both experiments.

**Fig 1:**
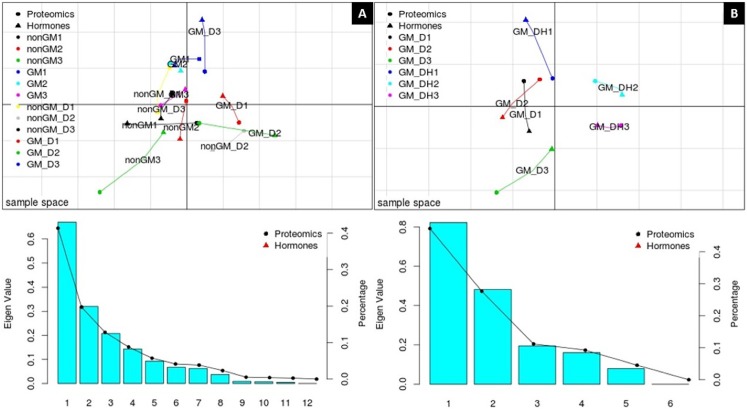
MCIA projection plot. (A) MCIA projection plot for Experiment 1, representing the proteomic and metabolomic datasets: PC1 = 41.35% and PC2 = 19.73%. (B) MCIA projection plot for Experiment 2, representing the proteomic and metabolomic datasets: PC1 = 47.29% and PC2 = 27.7%. PC1 is represented by the first axis (horizontal), and PC2 is represented by the second axis (vertical). Eigenvalue and Percentage graphics show the amount of variation in the dataset corresponding to each PC. Different symbols represent the respective “omics” analysis and are connected by lines where the length is proportional to the divergence between the data from a same replicate. Lines are joined by a common point, representing the reference structure, which maximizes covariance derived from the MCIA synthetic analysis. Colors represent the biological replicates.

The coordinates of each pool of biological replicates for each treatment are connected by lines, the length of which indicates the divergence (the shorter the line, the higher the level of concordance) between the hormone (and related compounds) and protein relative abundance levels for a particular replicate. For the Experiment 1 dataset (Fig 1A), the MCIA plot shows the two principal components (PC1 x PC2). PC1 separated the biological replicates of maize varieties submitted to drought stress from those of non-stressed varieties, accounting for 41.35% of the total variation in the dataset. PC2 separated GM from non-GM varieties, which accounted for 19.73% of the total variation. Fig 1B shows the MCIA plot for Experiment 2. In this case, the MCIA plot shows similar trends in proteome and metabolic profiles, indicating that the sources of biological information for most variants were similar. The MCIA plot also showed that both PC1 and PC2 separated samples with herbicide application from those whithout application. PC1 accounted for 47.29% of the variation in the dataset, while PC2 accounted for 27.70% of the variation. The combination of both PCs correspond to ~75% of the variation in the experiment, representing a high percentage of the total variation. Based on these results, we conclude that the main source of quantitative variation in our study originated from the environment (factor stress), followed by the hybrids (genetic modification factor). As expected for different growing conditions, these results agree with those of previous studies [2,4,39,40]. Mesnage et al. [13] used the same MCIA approach to determine the relationship of proteome and metabolome profiles of non-GM and GM (NK603) maize seeds. Overall, the authors showed that the GM transformation process was the major contributor to variation in the protein and metabolite profiles, followed by the spraying of herbicide [13]. Other multivariate analyses, such as Principal Component Analysis (PCA), have been used to demonstrate similarities in the quantity of proteins and metabolites between different conditions in multi-omics studies. However, unlike MCIA analysis, PCA has to be done separately for each different “omic” approach [1–3]. Therefore, when conducting gene, protein and metabolite expression analyses in the different maize hybrids MCIA could be a more reliable integrative tool in this case than PCA.

### 3.2. Differential proteomic analysis

Total protein content mean was 4.50 ± 0.14 mg.g-1 (fresh weight) of leaf material, which is considered a sufficient quantity for 2D-DIGE analysis. No significant difference was observed in the one-way ANOVA (P < 0.05) for the protein content mean between different growing conditions in Experiments 1 and 2. The number of spots in a two-dimensional protein gel is one of the most important components for a comparative proteomics analysis. Essentially, the number of spots present in the gels of each treatment correlates with comparative efficiency. Here, the average number of spots detected was 643 and, no statistically significant difference was found between or within treatments (plant varieties and growing conditions) in either experiment (one-way ANOVA, P < 0.05; data not shown). Comparative proteomic studies using 2D-DIGE analysis in plant leaves have shown an average number of detected spots ranging from 500 to 900, which agrees with our results [41–43].

Overall comparative analysis revealed a total of 20 different proteins that were present, absent, or up/downregulated in one of the treatments at statistically significant level (P < 0.05) (Tables 2 and 3). Proteins that were not detected in this study were below the detection limit of ± 1 ng and were therefore considered absent in the sample. All 20 different proteins were identified using Quadrupole Time-of-Flight (Q-TOF) tandem mass spectrometry analysis (MS/MS) (P < 0.05). Table 2 shows the Log2 fold-change variation of identified protein for each comparison. Table 3 shows a description of each of these proteins, including the associated biological process and molecular function according to the Uniprot database.

**Table 2:** Fold-change (Log2FC) of differentially abundant proteins in herbicide-tolerant GM maize NK603 and its non-GM near-isogenic variety grown under suboptimal conditions in Experiment 1 and 2. A positive value indicates that the protein showed up-regulation for the first treatment of the comparison. A negative value means that the protein showed downregulation for the first treatment of the comparison. ‘ns’ indicates that no significant difference (ANOVA P < 0.05) was found for the relative abundance of this protein on that specific comparison.

Protein	UniProt ID	Experiment 1				Experiment 2
		Factor 1 (Stress)		Factor 2 (Genetic Modification)		Unifactorial
		non-GM D x non-GM	GM D x GM	GM x non-GM	GM D x non-GM D	GM DH x GM D
ATP synthase subunit gamma, chloroplastic precursor (*Zea mays*)	P0C1M0	-1.34	-1.19	ns	ns	1.33
Ferredoxin-NADP reductase (*Zea mays*)	B6T9S5	0.57	-0.75	ns	-0.93	0.72
Photosystem I reaction center subunit II (*Zea mays*)	B6SKI1	0.71	ns	ns	-0.80	ns
Photosystem I reaction center subunit IV A (*Zea mays*)	B6SPC1	1.21	ns	ns	ns	ns
ATP synthase CF1 alpha subunit (*Saccharum* hybrid cultivar SP-80-3280)	A0A0U2U1F5	ns	-0.95	ns	ns	ns
ATP synthase CF1 beta subunit (chloroplast) (*Zea mays*)	P00827	ns	0.76	ns	ns	ns
NAD-dependent epimerase/dehydratase (*Zea mays*)	B4FH62	ns	-0.77	ns	ns	ns
Photosystem I subunit VII (chloroplast) (*Zea mays*)	P11601	ns	-0.81	ns	-0.84	ns
Oxygen-evolving enhancer protein 2, chloroplastic (*Zea mays*)	A0A096QKN7	ns	-0.85	ns	-0.67	-0.44
Phosphoglycerate kinase (*Zea mays*)	C0PDB0	ns	-0.96	ns	1.42	ns
Cytochrome b6-f complex iron-sulfur subunit (*Zea mays*)	B4FTU7	ns	0.63	ns	ns	ns
Inorganic pyrophosphatase (*Zea mays*)	B6SQQ0	ns	-0.65	ns	ns	0.57
psbP-like protein 2, chloroplastic (*Zea mays*)	A0A096TZ12	ns	ns	0.88	ns	ns
3-phosphoshikimate-1-carboxyvinyltransferase (*Glycine max*)	Q71LY8	ns	ns	1.26	1.17	Exclusive GM D
Ribulose bisphosphate carboxylase/oxygenase activase, chloroplastic precursor (*Zea mays*)	Q9ZT00	ns	ns	-0.80	ns	ns
Ribulose bisphosphate carboxylase large subunit (*Zea mays*)	A0A059Q6R9	ns	ns	-0.86	ns	ns
Chain A, The Crystal Structure of Psbp (*Zea mays*)	A0A096Q0E5	ns	ns	0.49	ns	ns
Malate dehydrogenase (*Zea mays*)	B4FZU8	1.15	ns	ns	ns	ns
Fructose-bisphosphate aldolase (*Zea mays*)	C0PD30	ns	ns	0.64	ns	ns
Uncharacterized protein LOC100273394 (*Zea mays*)	B4FV82	ns	-1.06	ns	ns	ns

**Table 3:** Description of differentially expressed proteins found in this study. Proteins were considered differentially modulated at statistically significant difference in normalized volume in each comparison between the treatments (GM; non-GM; GM D; non-GM D; GM DH) at ANOVA P < 0.05 according to Table 2. Proteins were classified in functional categories based on the KEGG Orthology databases and on careful literature evaluation. The table reports spot number (Match ID), accession number and protein name, together with Mascot score, sequence coverage, number of matched peptides, theoretical and experimental molecular weight (MW) and isoelectric point (pI).

Spot ID	Accession	Protein Name	Mascot Score	Sequence Coverage (%)	Peptides	Theor. Mass (kDa)	Theor. pI (pH)	Exp. Mass (kDa)	Exp. pI (pH)	Biological Process / Molecular Function
1689	NP_001150872	ATP synthase subunit gamma, chloroplastic precursor (*Zea mays*)	520	31	12	54	5.31	52	5.4	Energy metabolism / Stress response (ATP synthesis coupled proton transport)
1576	ACG33858	Ferredoxin-NADP reductase, (*Zea mays*)	536	44	19	40	8.56	44	8.2	Energy metabolism (oxidoreductase; nucleotide binding)
1364	ACG25364	Photosystem I reaction center subunit II (*Zea mays*)	220	24	5	21	9.77	20	9.1	Energy metabolism (photosynthesis)
1329	NP_001149700	Photosystem I reaction center subunit IV A (*Zea mays*)	204	47	6	14	9.79	15	9.1	Energy metabolism (photosynthesis)
1881	YP_024377	ATP synthase CF1 alpha subunit (*Saccharum* hybrid cultivar SP-80-3280)	842	40	17	55	5.87	55	5.8	Energy metabolism (ATP hydrolysis-coupled proton transport)
1826	NP_043032	ATP synthase CF1 beta subunit (chloroplast) (*Zea mays*)	237	16	5	54	5.31	55	5.4	Energy metabolism (ATP hydrolysis-coupled proton transport)
1511	ACG31416	NAD-dependent epimerase/dehydratase (*Zea mays*)	264	35	7	31	9.11	30	9.1	Carbohydrate metabolism (catalytic activity)
1261	NP_039445	Photosystem I subunit VII (chloroplast) (*Zea mays*)	110	46	3	8	6.51	8	6.2	Energy metabolism (photosynthetic electron transport in photosystem I; oxidoreductase)
1429	XP_008652004	Oxygen-evolving enhancer protein 2, chloroplastic (*Zea mays*)	345	37	8	31	9.50	33	9.1	Energy metabolism (photosynthesis; calcium ion binding; photosystem I)
1756	NP_001147628	Phosphoglycerate kinase (*Zea mays*)	413	23	10	50	6.07	48	5.8	Carbohydrate metabolism (glycolytic process; phosphorylation)
1365	ACG28186	Cytochrome b6-f complex iron-sulfur subunit (*Zea mays*)	204	28	5	24	8.52	21	8.4	Energy metabolism (photosynthesis; oxidoreductase activity; electron transport)
1529	ACG27183	Inorganic pyrophosphatase (*Zea mays*)	744	49	19	31	5.79	30	5.9	Carbohydrate metabolism (phosphate-containing compound metabolic process; magnesium ion binding)
1341	XP_008654301	psbP-like protein 2, chloroplastic (*Zea mays*)	301	36	8	25	8.34	25	7.5	Energy metabolism (photosynthesis; calcium ion binding; photosystem II)
1763	AAL67577	3-phosphoshikimate-1-carboxyvinyltransferase (*Glycine max*)	944	50	19	47	5.13	49	5.2	Energy metabolism (chorismate biosynthetic process; shikimate pathway);
1743	NP_001104921	Ribulose bisphosphate carboxylase/oxygenase activase, chloroplastic precursor (*Zea mays*)	487	31	12	47	6.29	49	6.4	Energy metabolism (Calvin cycle; ATP binding; activation of RuBisCO)
1848	NP_043033	Ribulose bisphosphate carboxylase large subunit (*Zea mays*)	521	27	12	52	6.33	52	6.3	Energy metabolism (photosynthesis; carbon fixation)
1449	4RTH_A	Chain A, The Crystal Structure of Psbp (*Zea mays*)	272	44	6	20	5.96	21	6.0	Energy metabolism (photosynthesis; calcium ion binding)
1638	NP_001142100	Malate dehydrogenase (*Zea mays*)	110	22	4	35	8.23	34	8.2	Carbohydrate metabolism (oxidoreductase)
1617	ACG33017	Fructose-bisphosphate aldolase (*Zea mays*)	522	27	10	40	5.39	39	6.1	Carbohydrate metabolism (glycolysis; carbohydrate degradation)
1581	NP_001141303	Uncharacterized protein LOC100273394 (*Zea mays*)	109	11	3	31	5.57	31	5.4	Unidentified

**Fig 2:**
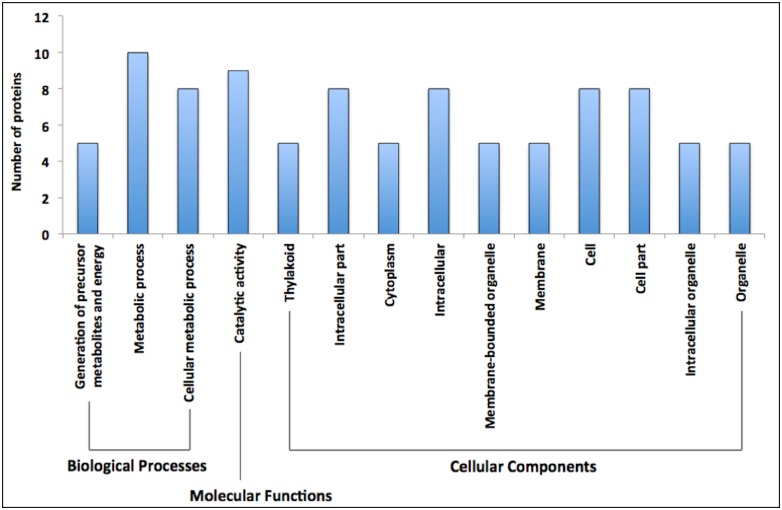
Enrichment analysis of 20 differentially expressed proteins of herbicide-tolerant GM maize variety (NK603) and its non-GM near-isogenic counterpart under control and stress conditions (drought and herbicide application). The analysis was performed using the online tool agriGO v1.2, using Single Enrichment Analysis (SEA) with the following parameters: 1) Selected species: *Zea mays* ssp; 2) Statistical test method: Hypergeometric; 3) Multi-test adjustment method: Hochberg (FDR); 4) Significance level of 0.05; 5) Minimum number of 5 mapping entries; and 6) Gene ontology type: Plant GO Slim.

We performed a pathway enrichment analysis in order to rank associations between the set of differentially regulated proteins representing metabolic pathways and the respective statistical probability. This analysis allows us to identify the relevant results of potentially altered pathways. All significantly enriched GO terms (FDR < 0.05) are shown in Fig 2. The altered biological processes with the highest number of assigned proteins were metabolic process (GO:0008152), catalytic activity (GO:0003824) and cellular metabolic process (GO:0044237). The differentially abundant proteins related to each different biological process will be discussed separately in the following sections.

#### 3.2.1. Experiment 1—Effects of drought stress

Two separate pairwise analyses were carried out to assess the effects of drought stress in GM and non-GM plants: GM D x GM and non-GM D x non-GM. A total of 14 differentially abundant proteins were observed in the two comparisons. Five of these proteins were differentially abundant in control only, with log2 fold-change values (log2FC) ranging from -1.34 to 1.15 (Table 2). Nevertheless, 4 of these 5 proteins were upregulated in the stress treatment, except for ATP synthase subunit gamma (chloroplastic precursor), which was downregulated in stress. These 5 proteins are involved in molecular functions belonging to energy metabolism and stress response biological processes (Table 3). The comparison between GM D x GM revealed 11 differentially abundant proteins with log2FC ranging from -1.19 to 0.76, and most of the proteins were downregulated in the GM D samples. These 11 proteins are not only involved in energy metabolism, but also in primary reactions of carbohydrate metabolism, such as glycolysis and phosphorylation (Table 3).

As expected, some of the proteins were found differentially abundant in both comparisons, but with distinct fold-changes. For instance, ATP synthase subunit gamma (chloroplastic precursor) was downregulated in the GM D samples (log2FC = -1.19) and in non-GM D samples (log2FC = -1.34), when compared to the control under optimal conditions. ATP synthase is an enzyme that modulates the electrochemical gradient across the thylakoid membrane and directly influences the rate of photosynthetic electron transport, thus representing a key factor in the regulation of photosynthetic energy conversion in chloroplasts [44]. Kohzuma et al. [45] demonstrated that thylakoid membranes of wild watermelon under drought conditions were drained of chloroplast ATP synthase e-subunit. Later, the same authors reported the downregulation of ATP synthase as a natural response of plants to drought stress [46]. In addition, Western blotting revealed that ATP synthase content decreased significantly in wild watermelon plants under drought stress. Therefore, these authors [46] also showed that plant responses to drought stress influence the regulatory ‘set point’ of photosynthesis by changing the expression of chloroplast ATP synthase and its activity. Thus, as an adaptive response, decreasing ATP synthase content leads to a more sensitive ‘set point’ for downregulation of the antenna, tending toward photoprotection over photosynthetic efficiency under stress such that the accessibility of light is likely to far transcend that for different resources needed to store photosynthetic vitality, e.g., water or CO_2_ [46]. Our results endorse these findings and suggest that quantitative control of ATP synthase is a consequence of plant response to the regulation of photosynthetic energy conversion under adverse environmental conditions.

On the other hand, Ferredoxin-NADP reductase showed different regulation in GM D samples (log2FC = -0.75) and non-GM D samples (FC = 0.57) when compared with the same varieties under normal growth conditions (Table 2). This protein is also located at the thylakoid membranes that participate in the ferredoxin reductase pathway responsible for NADPH production, which, in turn, is primarily used in the Calvin cycle [47]. Lehtimäki et al. [48] reported that the total expression level of the Ferredoxin-NADP+-oxidoreductase genes increased upon drought stress in *Arabidopsis thaliana*. In contrast, a decrease in Ferredoxin-NADP reductase was reported in *Populus cathayana* and wheat in response to drought stress [49], which seems to be the same case for the non-GM hybrid under drought stress. Most other differentially abundant proteins in the GM D x GM comparison were downregulated in the GM D treatment, such as ATP synthase CF1 alpha subunit, NAD-dependent epimerase/dehydratase, Photosystem I subunit VII (chloroplast), Oxygen-envolving enhancer protein 2 (chloroplastic), Phosphoglycerate kinase, Inorganic pyrophosphatase, and Uncharacterized protein LOC100273394. They are all involved in energetic metabolism and processes, including transport activities in photosynthesis, oxidoreductase, photosynthetic carbon metabolism and catalytic activities. Previous proteomic studies have reported similar results, showing that drought stress leads to an overall reduction of the photosynthetic transport chain [50], which consequently induces oxidative stress [51].

During the past few years, several reviews have discussed how drought constrains photosynthesis [52,53]. Although some controversy on the subject remains, decreased CO_2_ diffusion from the atmosphere to the site of carboxylation is generally considered the main cause for decreased photosynthesis under mild to moderate water limitation [52,54,55]. Corroborating our findings, other studies also recognize energetic metabolism as a key component of cellular function under limited supply of CO_2_ as a consequence of drought stress [53,56]. For instance, under these stress conditions, Tezara et al. [57] found impaired ATP production and, thus, ribulose bisphosphate regeneration. More recently, reactive oxygen species generated under highly reduced conditions in the chloroplast were shown to damage ATP synthase [53].

**Fig 3:**
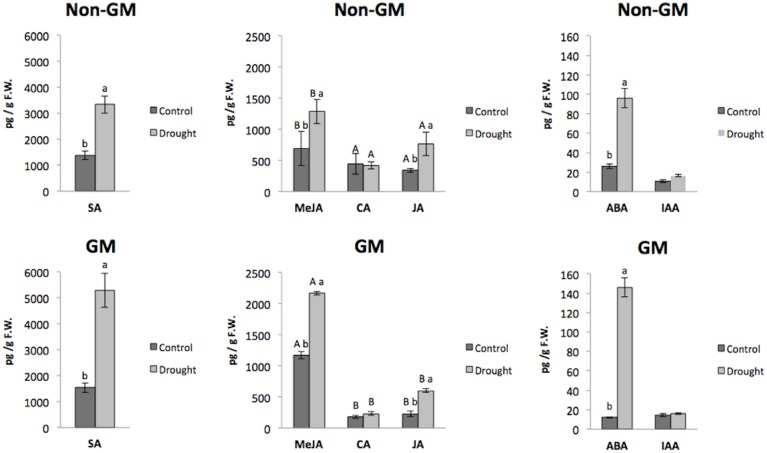
Levels of plant hormones and related compounds for Experiment 1. Levels of SA, MeJA, CA, JA, ABA and AIA in leaves of herbicide-tolerant GM maize (NK603) variety and its non-GM counterpart under control and drought stress conditions. Compound levels were considered significantly different at ANOVA *P* < 0.05. When significant, lowercase letters represent the horizontal comparisons in each compound from the Stress factor (non-GM D x non-GM; GM D x GM). Uppercase letters represent the vertical comparisons from the Genetic Modification factor (GM x non-GM; GM D x non-GM D)

Regarding to the phytohormone and related compounds analysis, our results showed statistically significant difference in four (SA, MeJA, JA and ABA) of the six phytohormones and related compounds quantified (Fig 3). All four hormones revealed average levels significantly higher in the treatments under drought stress (non-GM D and GM-D) compared to control treatments (non-GM and GM), showing log2FC values between 0.89 (MeJA) and 3.60 (ABA) (Table 4). These results were expected since these four plant growth regulators have all been previously associated with plant response to environmental changes via cell signaling pathways [58].

**Table 4:** Fold-change (Log2FC) for plant hormones and related compounds levels in herbicide-tolerant GM maize variety (NK603) and its non-GM near-isogenic variety in different comparisons of Experiment 1 and 2. A positive value indicates that the compound showed upregulation for the first treatment of the comparison. A negative value indicates that the compound showed downregulation for the first treatment of the comparison. ‘ns’ indicates that no significant difference was found (ANOVA P < 0.05) for the levels of this compound on that specific comparison.

Hormone	PubChem ID	Experiment 1				Experiment 2
		Factor 1 (Stress)		Factor 2 (Genetic Modification)		Unifactorial
		non-GM D x non-GM	GM D x GM	GM x non-GM	GM D x non-GM D	GM DH x GM D
Salicylic Acid (SA)	338	1.27	1.78	ns	ns	-1.35
Methyl Jasmonate (MeJA)	5367719	0.89	0.89	0.75	0.75	ns
Cinnamic Acid (CA)	444539	ns	ns	-1.28	-0.85	ns
Jasmonic Acid (JA)	5281166	1.15	1.37	-0.56	-0.34	1.01
Abscisic Acid (ABA)	5280896	1.88	3.60	ns	ns	2.92
Indole-3-acetic acid (IAA)	801	ns	ns	ns	ns	ns

Water deficit is widely known to affect biosynthesis, accumulation, and redistribution of major plant hormones and other related compounds. ABA, which is synthesized either in leaves or in roots, plays a major role in commanding stomatal closure and, therefore, photosynthetic carbon uptake under conditions of water deficit [59,60]. In addition, ABA synthesis is one of the fastest responses of plants to drought stress, which triggers ABA-inducible gene expression [61], causing stomatal closure, thereby reducing water loss via transpiration [62] and eventually restricting cellular growth. Nevertheless, recent studies have shown evidence of crosstalk between different hormones and other compounds, such as auxin, cytokinin, ethylene, brassinosteroids, jasmonates, and salicylic acid, resulting in synergistic or antagonistic interactions and coordinated regulation of hormone biosynthetic pathways, which play crucial roles in the response of plants to abiotic stress [31,63–65].

Acharya and Assman [66] suggested that other hormones, such as JA, SA and IAA, are also involved in stomatal function. Whereas ABA, SA, JA and other related compounds induce stomatal closure, IAA, together with citokinin, promotes stomatal opening. These hormones act to modulate the expression of different drought-related genes [67,68]. A wide range of cellular biochemical events has also been associated with the regulation of stomatal guard cells, such as the activation of G-proteins, the production of reactive oxygen species (ROS) (ABA-stimulated), protein phosphorylation/dephosphorylation, and reorganization of the cytoskeleton [66]. Under drought stress, our results showed a significant increase in ABA levels in GM plants, as well as a significant downregulation of proteins involved in phosphorylation and dephosphorylation, such as Phosphoglycerate kinase and Inorganic pyrophosphatase. Phosphoglycerate kinase catalyses the phosphorylation of 3-PG, producing 1,3-BPG and ADP, as part of the reactions that regenerate ribulose-1,5-bisphosphate in the Calvin cycle. On the other hand, inorganic pyrophosphatases are important enzymes that generate the thermodynamic driving force for some cellular biosynthetic reactions by catalysing the hydrolysis of inorganic pyrophosphate (PPi) to inorganic orthophosphate (Pi) [69]. Pyrophosphatases not only play a crucial role in ATP production, but are also important for biopolymer synthesis in the construction of new cell walls and membranes [70,71]. Similar to our results, Fukuda and Tanaka [72] showed that ABA mediates the expression of genes responsible for regulating pyrophosphatases in response to osmotic stresses resulting from environmental changes, e.g., salt and drought stress.

#### 3.2.2. Experiment 1—Effects of genetic modification

The comparative analysis between genetically modified and unmodified varieties: GM x non-GM and GM D x non-GM D, which was performed to assess the effects of genetic modification, revealed a total of 11 differentially regulated proteins. The GM x non-GM comparison showed six differentially abundant proteins, with four of them upregulated in the GM variety, and these four are involved in carbohydrate and energy metabolism: psbP-like protein 2 (chloroplastic), EPSPS, Chain A, and Fructose-bisphosphate aldolase (Table 2). Proteins involved in carbohydrate and energy metabolism have also been detected in other proteomic studies comparing GM varieties and their near-isogenic conventional counterparts [1–4,73–76]. These results suggest that GM crops have a higher energy demand in comparison to conventional plants which do not bear the necessity of calling upon energy sources to synthetize transgenic proteins. In addition, previous findings suggested that the strong and constitutive promoters, e.g., CaMV-35S, used for genetic modification could lead to this high energetic cost in transgenic plants [77,78]. If so, additional studies should be carried out to identify which metabolic pathways will be more affected by the reduced amount of available energy.

The other two proteins detected, Ribulose bisphosphate carboxylase/oxygenase activase and Ribulose bisphosphate carboxylase large subunit, showed downregulation in the GM variety. These are two similar proteins, located in the chloroplast, and they play major roles in the activation of RuBisCO activity. The drop in Rubisco activase is presumably a key factor in slowing down Rubisco activity [53]. Downregulation of key enzymes involved in RuBisCO activity might result in a reduction of photosynthetic rates of the GM plants (NK603). Since none of these six differential proteins from the GM x non-GM comparison was differentially abundant for factor 1 (stress) comparative analysis, such reduction in photosynthetic rates could be a result of genetic modification. Again, it remains to be scrutinized if this reduction of photosynthetic rates will impose additional burden on plant development.

The GM D x non-GM D comparison also showed six differentially abundant proteins, with four downregulated in the GM D treatment. Again, these are proteins that play crucial roles, including, for example, electron transport and oxidoreductase activity, in photosynthesis, acting mainly in Photosystem I. On the other hand, drought stress leads to a reduction of the photosynthetic rates, but in the present study, GM plants have shown a significant downregulation of these photosynthesis-related proteins compared to their non-GM near-isogenic variety under the same environmental stresses.

MeJA, CA and JA showed significantly different levels between the GM and its non-GM near-isogenic variety under both control and stress conditions (Fig 3). MeJA levels increased in the GM and GM D samples, while significantly lower levels were observed for CA and JA. Opposite regulation of JA and MeJA was an unexpected result since they are synthesized in the same main octadecanoid pathway. Moreover, these compounds play important roles together to activate plant defense mechanisms in response to biotic and abiotic stresses, such as drought, low temperature, and salinity [79].

The aromatic amino acid products of the shikimate pathway (phenylalanine, tyrosine and tryptophan) are not only essential components of protein synthesis, but also serve as precursors for a wide range of secondary metabolites that are important for plant growth. Sasaki-Sekimoto et al. [80] reported that the Arabidopsis gene encoding IGPS of Tryptophan biosynthesis is regulated by JA. Since the shikimate pathway is upregulated in GM plants by the insertion of a new EPSPS, our results suggest that JA levels in herbicide-tolerant GM plants are higher from the possible upregulation of the shikimate pathway to increase tryptophan production.

CA is also a key intermediate in the shikimate pathway, and it is formed through deamination of L-phenylalanine, which is one of the primary aromatic amino acid products of the shikimate pathway [81]. Since the NK603 GM event was modified to produce a glyphosate-based herbicide-tolerant form of EPSPS enzyme, which is also involved in the biosynthesis of phenylalanine, tryptophan and tyrosin in the shikimate pathway, our findings suggest a possible influence of the genetic transformation on the synthesis of secondary nontarget metabolites as result of new pleiotropic effects by the presence of the integrated transgene.

Overall, the differences in the protein profile and metabolite levels between the GM maize NK603 event and its near-isogenic non-GM variety can affect many physiological processes and biochemical pathways, such as photosynthesis-related pathways. Taken together, our results demonstrate that these two hybrids may not behave similarly at the molecular level, thus precluding the assumption that they are substantially equivalent.

#### 3.2.3. Experiment 2—Combined effects of drought and herbicide stress

In the second experiment, we tested the influence of herbicide application on plants under drought stress, and we compared GM DH x GM D samples. The comparative proteomic analysis revealed four differentially abundant proteins differentially, three of which were shown to be upregulated in the GM DH samples (Table 2). These proteins were associated with increased energy metabolism, such as ATP synthase subunit gamma, Ferredoxin-NADP reductase and Inorganic pyrophosphatase, suggesting that an increased amount of energy is required to adapt to cumulative stress. Meanwhile, the downregulation of Oxygen-envolving enchancer protein 2 (chloroplastic) might be a result of photosynthesis reduction in these plants under drought and herbicide stress, resulting from involvement in the photosynthesis process. Ahsan et al. [82] found similar results when analysing the proteomic profile of rice leaves under two concentrations of arsenate. An increased activity of several proteins associated with energy metabolism was found, confirming the assumption that energy is required for stress adaptation. Glyphosate-based herbicide was also applied to rice leaves as a stress factor, and it resulted in increased accumulation of antioxidant enzymes, such as ascorbate peroxidase, glutathione S-transferase, thioredoxin h-type, nucleoside diphosphate kinase 1, peroxiredoxin and a superoxide dismutase [Cu—Zn] chloroplast precursor. Therefore, the authors suggest that glyphosate-based herbicides might induce oxidative stress in plants [13, 83].

Furthermore, the comparative analysis showed exclusive expression of the EPSPS synthase protein in plants without herbicide application (GM D), which was expected since native EPSPS synthase is inhibited by the glyphosate-based herbicide. This is an important enzyme involved in the shikimate pathway, which is primordial in one step of chorismate synthesis, preceding the synthesis of some essential aromatic amino acids, such as phenylalanine, tyrosine and tryptophan [84]. In addition, this pathway inhibited by the herbicide based on Glyphosate (N-(phosphonomethyl)glycine) is also involved in the synthesis of lignin, flavonoids, phenolic compounds, and other secondary metabolites that can account for as much as 35% of a plant’s biomass [85].

**Fig 4:**
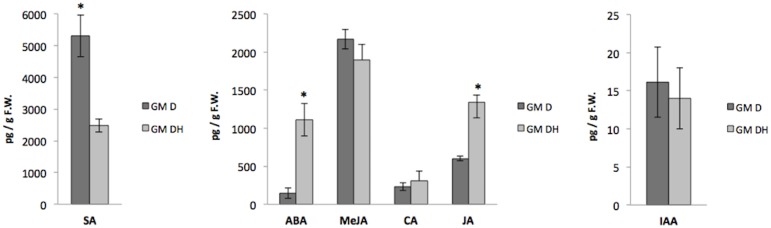
Levels of plant hormones and related compounds for Experiment 2. Levels of SA, ABA, MeJA, CA, JA, and AIA in leaves of herbicide-tolerant GM maize NK603 under drought stress conditions and herbicide application. (*) Compound level was considered significantly different at ANOVA *P* < 0.05.

Comparison of GM D x GM HD revealed that sequential applications of glyphosate-based herbicide resulted in different levels of SA, ABA and JA (Fig 4). While SA levels were significantly lower in the GM HD samples, ABA and JA were detected at higher levels in the same treatment, when compared to the absence of the herbicide. Plants that received herbicide application showed levels of ABA and JA up to 8 times greater (log2FC = 2.9) than those without herbicide (Table 4). Interestingly, proteomic analysis showed that an ATP synthase subunit gamma protein was upregulated in the GM DH samples. This protein mediates plant perception in stress through the induction of volatile, phenylpropanoid and protease inhibitor defenses, such as JA and SA [86].

Doğramacı et al. [87] studied the impact of glyphosate-based herbicide on the vegetative growth of Leafy spurge (*Euphorbia esula*), and although abundance of jasmonic acid (JA) was not quantified, significant increase of transcripts homologous to Arabidopsis genes involved in biosynthesis of JA was also observed in plants treated with the herbicide. The authors also reported a significant increase in other important bioactive auxins (IAA, IBA) in glyphosate-based, herbicide-treated plants.

Reduced amounts of EPSPS caused by the glyphosate-based herbicide led to reductions in aromatic amino acids and indole-3-acetic acid (IAA), which is an important signaling hormone for plant growth and development, ultimately causing the plant to weaken and die [88]. Although not statistically significant, a decrease of IAA levels was verified in the GM samples treated with herbicide (GM DH). This was not expected since these plants constantly synthesize a substantial equivalent EPSPS enzyme. Although these plants were genetically modified to produce a glyphosate-tolerant form of EPSPS, the herbicide is somehow affecting the secondary metabolism of these herbicide-tolerant plants. Consequently, the synthesis of other compounds, such as lignin, flavonoids, phenolic compounds, and other secondary metabolites, is also involved in the shikimate pathway and may also be altered as an unintended effect. In addition, Bohn et al. [9] reported that herbicide-tolerant GM soy may have high residue levels of glyphosate and aminomethylphosphonic acid (AMPA) and that different agricultural practices may result in a markedly different nutritional composition of soybeans. Thus, in addition to presenting a markedly different nutritional composition and gene expression, secondary metabolism could be affected by the presence of the transgene and/or Roundup herbicide.

Moreover, it is important to mention that the NK603 event carries two gene cassettes, both containing the CP4 EPSPS coding sequence, but under the regulation of different promoters. While the first one contains the 5'-end of the rice actin sequence (ract1) promoter and the first intron upstream of the CTP sequence, the second CP4 EPSPS gene is under the control of the enhanced CaMV 35S promoter fused to the 0.8 kb intron sequence from the gene of maize heat shock 70 protein [89]. Castan et al. [90] recently reported a study in which the NK603 construct was sequenced, with the aim of examining its genetic stability. The authors consistently detected two nucleotide insertions in all sequenced samples of NK603 × MON810 (varieties 631RR2/Bt and DKC 26–79 progeny), as well as the certified reference material, by comparing the sequenced construct NK603 to the published patent sequence [91]. Since the detected nucleotide insertions were located in a promoter region, the authors highlighted the possibility of an influence on promoter activity and, hence, the expression rate of the transgene. However, determining whether one of the cassettes was the major source of the impacts affecting proteins and hormones in unexpected ways is outside the scope of the present paper, but we could consider this hypothesis for future specific studies.

### 3.3. Contributions to risk assessment

Our findings challenge the general presumption of molecular stability of commercially approved GM plants compared to their non-GM near-isogenic varieties, in particular when considering environmental variables. Although no consensus has been reached on the overall utility of omics techniques, they have been acknowledged as a potentially valuable tool in RA [92–95]. Trough the use of omics analysis, our study has shown that proteins assigned to important biological processes, such as energy/carbohydrate metabolism, have had their relative abundance level altered in GM plants under normal conditions and abiotic stresses and this might have a safety relevance. The applicability and usefulness of proteomics and metabolomics to the molecular characterization step of premarket biosafety RA of GMOs is still an evolving view, many studies, including the present one, demonstrate the feasibility and the necessity of such characterization studies for any GM event. In addition, as new kinds of GMOs are developed, wide use of molecular profiling should be added to the required criteria for risk assessment.

Even though studies have assessed unintended effects of GM plants derived from genetic modification based on “omics” approaches, most of them still evaluate these plants under controlled or invariable growth conditions [1,73,95]. The aim of the present study was to broaden knowledge about potential variations in the proteome and secondary metabolism of GM plants that result from genetic transformation, environmental variables, or the interaction between these factors. So far, no similar study has integrated proteomics and metabolomics approaches to explore plant molecular response of GM plants to abiotic stresses in a manner equal to that of the present work.

It is anticipated that the present work will pave the way for collaborative studies for the development of gene, protein, and metabolite databases of important crops grown across a range of environmentally variable conditions, thus providing an important and useful benchmark for safety assessment [92]. It is also hoped that this work will set a precedent whereby newly developed GM plants are subjected to experimentation under different environmental conditions as a part of risk assessment before approval by regulatory agencies.

## 4. Conclusions

Significant differences in protein relative abundance and levels of plant hormones and related compounds of the herbicide-tolerant GM NK603 maize variety under optimal and stress conditions were observed. The protein profile of GM maize under different environmental conditions revealed 20 different proteins, which were classified into three major categories: metabolic process, catalytic activity and cellular metabolic process, with most of the detected proteins assigned to energy/carbohydrate metabolic processes. Our findings suggest that environmental factors, such as drought and herbicide application, are major sources of quantitative variation in protein relative abundance and phytohormones/related compound levels, followed by the genetic modification. Some differences in protein relative abundance (11 proteins) and some compound levels (JA, MeJA and CA) were found between the GM plant and its non-GM near-isogenic variety under the same environmental conditions, suggesting that genetic modification itself may also play an important role in the appearance of pleiotropic effects. Taking into account the consequences of differences in protein profile and metabolite levels, including hormones and related compounds, the results found in this study do not support the substantial equivalence between the tested GM maize (NK603) and its non-GM near-isogenic variety.

Therefore, based on environmental variables, especially those exerting stress, it was our goal to show that proteomic profiling can assist in the detection of unintended changes that cannot otherwise be detected using a comparison with field-grown plants under suboptimal conditions. Our research findings could be applied to the GMO RA step right away; therefore, they are likely to be of great interest to scientists, researchers, regulators, industrial leaders, and other private organizations involved in the development and/or evaluation of GMO safety.

## Supporting information

S1 File Metabolomic analysis supplementary data. Optimization of each analyte for Multiple Reactions Monitoring (MRM) transition and overlaid multiple reaction monitoring chromatograms of both positive and negative ionization mode.
(DOCX)

S2 File Supplementary protein data. Sequences of all peptides from the differentially abundant proteins identified.
(XLSX)
