# Supplementary material for: Molecular responses of genetically modified maize to abiotic stresses as determined through proteomic and metabolomic analyses
Source: PLoS One. 2017 Feb 28;12(2):e0173069. doi: 10.1371/journal.pone.0173069 (PMC5330488; doi:10.1371/journal.pone.0173069)
Supplement: S1 File — Optimization of each analyte for Multiple Reactions Monitoring (MRM) transition and overlaid multiple reaction monitoring chromatograms of both positive and negative ionization mode. (DOCX) [file pone.0173069.s001.docx]

**S1 File.** **Metabolomic analysis supplementary data.** Optimization of each analyte for Multiple Reactions Monitoring (MRM) transition and overlaid multiple reaction monitoring chromatograms of both positive and negative ionization mode.

| Name | **Ionization**  **mode** | **Precursor ion (m/z)** | **Product ion**  **(m/z)** | **Fragmentor (V)** | **CE**  **(V)** |
| --- | --- | --- | --- | --- | --- |
| Zeatin (Z) | **+** | **220** | **136** | **90** | **12** |
| Indole-3-acetic acid methyl ester (MeIAA) | **+** | **190** | **130** | **55** | **8** |
| Jasmonic acid methyl ester (MeJA) | **+** | **225.1** | **151** | **75** | **4** |
| Salicylic acid methyl ester (MeSA) | **+** | **153** | **120** | **55** | **0** |
| Cinnamic acid (CA) | **-** | **147** | **103** | **84** | **4** |
| Gibberellic acid (GA3) | **-** | **345** | **143** | **110** | **16** |
| Indole-3-acetic acid (IAA) | **-** | **174** | **130** | **62** | **4** |
| Salicylic acid (SA) | **-** | **137** | **93** | **80** | **12** |
| Abscisic acid (ABA) | **-** | **263** | **153** | **90** | **0** |
| Jasmonic acid (JA) | **+** | **209** | **59** | **82** | **4** |
| Jasmonic acid Isoleucine (JA-Ile) | **+** | **322** | **130** | **72** | **12** |
| 2-acetamidophenol (internal Standard) | **+ or -** | **152(+) 150(-)** | **110(+) 108(-)** | **72(+) 67(-)** | **8(+) 8(-)** |

Figure: Overlaid multiple reaction monitoring chromatograms of both positive and negative ionization mode.
